# Second-Order Conditioning and Conditioned Inhibition: Influences of Speed versus Accuracy on Human Causal Learning

**DOI:** 10.1371/journal.pone.0049899

**Published:** 2012-11-28

**Authors:** Jessica C. Lee, Evan J. Livesey

**Affiliations:** School of Psychology, University of Sydney, Sydney, New South Wales, Australia; University of New South Wales, Australia

## Abstract

In human causal learning, excitatory and inhibitory learning effects can sometimes be found in the same paradigm by altering the learning conditions. This study aims to explore whether learning in the feature negative paradigm can be dissociated by emphasising speed over accuracy. In two causal learning experiments, participants were given a feature negative discrimination in which the outcome caused by one cue was prevented by the addition of another. Participants completed training trials either in a self-paced fashion with instructions emphasising accuracy, or under strict time constraints with instructions emphasising speed. Using summation tests in which the preventative cue was paired with another causal cue, participants in the accuracy groups correctly rated the preventative cue as if it reduced the probability of the outcome. However, participants in the speed groups rated the preventative cue as if it increased the probability of the outcome. In Experiment 1, both speed and accuracy groups later judged the same cue to be preventative in a reasoned inference task. Experiment 2 failed to find evidence of similar dissociations in retrospective revaluation (release from overshadowing vs. mediated extinction) or learning about a redundant cue (blocking vs. augmentation). However in the same experiment, the tendency for the accuracy group to show conditioned inhibition and the speed group to show second-order conditioning was consistent even across sub-sets of the speed and accuracy groups with equivalent accuracy in training, suggesting that second-order conditioning is not merely a consequence of poorer acquisition. This dissociation mirrors the trade-off between second-order conditioning and conditioned inhibition observed in animal conditioning when training is extended.

## Introduction

In a typical human causal learning experiment, cues are presented that may increase or decrease the likelihood of a particular outcome and the participant’s task is to assess to what degree each cue either causes or prevents that outcome. Various authors have suggested that this process involves elementary associative learning mechanisms because results from many of these experiments bear strong resemblance to animal conditioning phenomena (e.g. [1], [2]). Conditioned inhibition – or learning about a cue that has a negative contingency with an outcome – is one such example.

Conditioned inhibition results from experience with a feature negative (FN) discrimination, where one cue leads to an outcome (A+), but when it is paired with a second cue, no outcome occurs (AX−). After sufficient training with these contingencies, the test stimulus (X) typically acquires inhibitory properties, such that its presence reduces responding in animal conditioning [3] or lowers ratings of causation or contingency in human learning [4]. In other words,×becomes a conditioned inhibitor as a consequence of its negative contingency with the outcome. When paired with another cue that has previously signaled the outcome (e.g. B+) the conditioned inhibitor reduces behavioral anticipation of the outcome that would normally be elicited by B (i.e. a summation test; [3]). In human causal learning, some doubt has been cast over several experiments that purport to show conditioned inhibition because of the choice of appropriate controls (see [5]). However, several experiments have found evidence of conditioned inhibition using a conservative test in which the ratings for the critical summation test compound BX are compared to ratings for a compound of B and a neutral or novel stimulus [5], [6]. In these studies, ratings for BX were substantially diminished, indicating that learning about×reduces causal ratings above and beyond what would be expected from a simple external inhibition effect; the reduction in ratings produced by pairing B with any other stimulus that has not been paired with the outcome [5]. Thus, like several other phenomena, conditioned inhibition appears to be common to a range of very different learning paradigms from Pavlovian conditioning to human causal judgment.

The general conclusion that human judgments of causation have an associative basis has been challenged on several grounds, including parsimony [7]. Humans display cognitive abilities such as deductive reasoning (e.g. [8]) and rule abstraction [9], [10] that could succinctly explain many of the causal learning results without recourse to primitive learning mechanisms. The task of separating the contributions of associative learning from other forms of cognition is made difficult by the fact that most experimental results in causal reasoning and contingency judgement are consistent with multiple explanations. Conditioned inhibition, for instance, could be explained as the formation of an inhibitory link between the conditioned inhibitor×and the outcome, which negates excitatory associations between other cues and the outcome. Such explanations follow naturally from the mechanisms described in many associative learning models (e.g. [11]). However, alternatively one could interpret this as the participant forming an inference that cue×prevents the occurrence of the outcome [12]. These explanations are by no means mutually exclusive but both effectively account for the learned properties of the conditioned inhibitor.

Given this general problem of dissociating psychological processes from one another, the FN paradigm is particularly interesting because under some circumstances, the cue (X) that possesses a negative contingency with the outcome actually appears to acquire excitatory rather than inhibitory properties [13], [14], [15]. This effect is often referred to as second-order conditioning because×acquires excitatory properties via its association with an excitatory cue (A) that is paired directly with the outcome. Several animal learning studies suggest that a transition from second-order conditioning to conditioned inhibition occurs through the course of training, with inhibition developing slowly. For example, Yin, Barnet, and Miller [15] have shown that conditioned inhibition manifests only with extended training with the FN contingencies intermixed, while second-order conditioning is evident with fewer training trials, presented either interspersed or in a blocked (A+ then AX−) design.

Second-order conditioning is noteworthy because normative and inferential models predict that×should not be treated as a cause of the outcome, given its negative contingency (X never appears with the outcome). For this reason, the mere fact that second-order conditioning occurs is viewed as being potentially diagnostic of the psychological mechanisms involved in learning [16]. Evidence for this effect in human causal learning can be found in a study reported by Karazinov and Boakes [17], who found second-order conditioning by limiting participants’ time to think on each trial. Each participant completed a causal scenario in which they played the role of a doctor attempting to discover which foods consumed by a fictitious patient were causing migraine headaches. Participants in one group completed the training phase of the experiment in a self-paced fashion (as is usually the case in causal learning tasks), whereas another group were limited to three seconds to respond on each training trial. Embedded amongst several other contingencies, the participants were given a FN discrimination (P+/PX−), where the addition of×to P prevented a migraine from occurring. However, instead of judging the test stimulus (X) to be preventative of the outcome, as did the self-paced group, in both experiments the paced group gave the test cue a higher causal rating than they did a non-causal control cue (M) trained in compound (LM−). Results from the typical summation tests – comparing×to M in compound with a trained excitor (T+) – suggested a similar pattern. Experiment 1 revealed a group interaction whereby TX was rated higher than TM in the paced group, but neither conditioned inhibition nor second-order conditioning was evident in the unpaced group. In Experiment 2, the unpaced group rated TX lower than TM (consistent with conditioned inhibition) but no group interaction was evident and the paced group did not rate TX higher than TM.

Shanks ([16]; see also Mitchell et al., [7]) has recently cited this result as a compelling example of causal learning taking a form that defies any obvious explanation in terms of rational inference, suggesting instead the operation of associative processes in human causal learning. The result is particularly noteworthy because effects indicating excitatory and inhibitory learning were revealed with training on the same contingencies, albeit not within the same experiment. Other cue competition effects are known to be sensitive to the conditions of learning in a seemingly similar fashion. These include retrospective revaluation effects (e.g. mediated extinction versus release from overshadowing; [18]) and the evaluation of a redundant cue (e.g. blocking versus augmentation; [19]), which will be briefly discussed in relation to Experiment 2. However, by and large, studies rarely observe cue contingency effects of this nature occurring in both excitatory and inhibitory directions on the basis of a single manipulation. Karazinov and Boakes’ [17] results constitute the best evidence for a non-rational second-order conditioning effect in human causal learning. However, even in their study, excitatory and inhibitory simple effects were not found in the same experiment. The potential significance of the effect and the somewhat equivocal nature of Karazinov and Boakes’ result make it all the more important to replicate this dissociation and to examine its properties.

The primary aim of this study was to garner further evidence for Karazinov and Boakes’ [17] dissociation in the FN paradigm by varying additional training parameters in addition to their pacing manipulation, providing a stronger impetus to respond either as quickly or as accurately as possible. However, unlike Karazinov and Boakes, we wished to obtain the dissociation using an identical set of test stimuli to find effects consistent with conditioned inhibition and second-order conditioning. Both experiments used a between-subjects design to manipulate trial time (unpaced versus paced trials), accompanied by instructions and feedback that emphasized the importance of either accuracy or speed during learning. Participants given self-paced trials and instructions to be as accurate as possible were expected to show learning consistent with conditioned inhibition, as has been observed in similar causal learning tasks previously (e.g. [4]). Participants given trial time limits and instructions emphasizing speed were expected to show second-order conditioning, consistent with Karazinov and Boakes’ [17] findings. In each experiment, participants assumed the role of a pharmaceutical researcher learning about the effects of different drugs that could cause potential side-effects. The cues were novel drug names (e.g. Slevoral, Melixil), and the possible outcomes were the occurrence of migraine (Experiments 1 and 2), nausea (Experiment 1 only), or no outcome. Experiment 1 focused on the feature negative contingencies in a complex causal learning task involving multiple outcomes. Experiment 2 examined the effect of trial time restriction on other cue contingency effects in addition to the FN discrimination. To test the claim that normative and inferential models do not predict second-order conditioning [17], an inference test in Experiment 1 aimed to show that conditioned inhibition was the rational judgement that should have resulted in the speed group.

## Experiment 1

Experiment 1 primarily aimed to dissociate excitatory and inhibitory learning resulting from acquisition of the FN discrimination, using instructions, feedback and trial time limits to emphasise either speed or accuracy during training. In addition to the stimuli directly involved in the FN paradigm, other stimuli were included to assess transfer of learning and to function as filler cues (Table 1). The experiment used a scenario in which two possible side effects could occur as outcomes. Thus, each trial type was associated with “migraine”, “nausea”, or “no outcome”. Each participant completed two sets of FN discrimination and related control trials, one set involving migraine as the potential outcome, the other involving nausea (see Table 1).

**Table 1:** Cues and outcomes used in the training phase of Experiment 1.

Stimuli Set 1		Stimuli Set 2		Function
Cue	Outcome	Cue	Outcome	
A_1_	1	A_2_	2	Feature negative stimuli
A_1_X_1_	No O	A_2_X_2_	No O	
B_1_	1	B_2_	2	Controls used for summation test comparing BX to BC
C_1_	No O	C_2_	No O	
P_1_	No O	P_2_	No O	Fillers
Q_1_R_1_	1	Q_2_R_2_	2	

Outcome 1 was migraine, outcome 2 was nausea.

After training, both groups were given a self-paced ratings test, in which they were shown drug cues (or combinations of cues) and had to indicate the degree to which they expected each of the two side-effects to occur. The ratings test yielded two kinds of scores: outcome-specific ratings (specifically using the rating for the associated outcome during training) and the ratings difference scores (the difference between the ratings for the associated outcome and the alternative outcome). For example, the outcome-specific score for A_1_ was the rating for outcome 1 only, and the difference score was obtained by subtracting the rating for outcome 2 from the rating for outcome 1. The difference scores were included as a means of gauging outcome specificity in learning, allowing for learning that “X causes/prevents O1” to be distinguished from the generalised learning of “X causes/prevents a side-effect”, which would manifest as a change in ratings for both scales (e.g. see [20]).

To assess learning, non-causal cues C_1_ and C_2_ were combined with trained excitors (B_1_ and B_2_) to form a novel control compound, which would then be compared with a novel compound consisting of the test cues (X_1_ and X_2_) and the same trained excitors (B_1_ and B_2_). Thus, the presence of conditioned inhibition or second-order conditioning was assessed via a summation test by comparing these critical test stimuli B_1_X_1_ and B_2_X_2_, to controls B_1_C_1_ and B_2_C_2_. If participants had genuinely learned that the test stimuli (X_1_ and X_2_) were inhibitors, they should rate the probability of their respective illnesses occurring as being low when they are paired with different excitors, compared to when the excitors are paired with the non-causal (but also non-preventative) control cues (C_1_ and C_2_). This was thought to be a conservative but necessary measure of conditioned inhibition, since it is known that combining a trained excitor with another stimulus results in lower predictive ratings due to reasons other than conditioned inhibition (see [5], [6], [21]). Since the aim was to obtain the group interaction on the same test cues, the choice of control cue was driven by the need to compare excitatory and inhibitory learning with an unambiguously non-causal cue.

Conversely, a higher rating for BX than for BC indicates second-order conditioning has occurred as it suggests that the presence of×has an excitatory rather than an inhibitory relationship with the outcome. This is an atypical measure for second-order conditioning, which has conventionally involved testing individual stimuli. However, it is appropriate in this case for two reasons. First, both BX and BC are novel compounds and any effect on ratings generated by uncertainty about new combinations of drugs will affect both. Second, it provides a direct comparison with the evidence for conditioned inhibition. By any conventional analysis based on associative learning principles, the excitatory strength of B should not inflate ratings of BX any more than BC and thus if BX receives a higher rating than BC, it should be based on the participant’s evaluation of×vs. C. Following from both the animal literature and Karazinov and Boakes’ [17] results, it was expected that conditioned inhibition would be evident in the accuracy group. The question of most interest was whether this effect would interact with the group manipulation and, more specifically, whether second-order conditioning would occur in the speed group, where the opportunity to reflect on each trial is restricted. A self-paced inference test at the end of the experiment sought to clarify whether conditioned inhibition was considered a rational judgement, and specifically, whether the speed group would still show second-order conditioning when given the opportunity to reason about the contingencies.

### Method

#### Participants

Fifty-two first-year psychology students from the University of Sydney participated in exchange for partial course credit. Five participants who scored below 35% (slightly above chance) accuracy for the feature negative stimuli (mean of A_1_, A_2_, A_1_X_1_ and A_2_X_2_) in the last quarter of the training phase were excluded, leaving 23 participants in the speed condition, and 24 in the accuracy condition (37 female, mean age = 19.8 years). All participants gave written informed consent and the procedure was approved by the University of Sydney Human Research Ethics Committee.

#### Apparatus

The experiment was programmed using Psych Toolbox for Matlab [22], [23] and run on Apple Mac Mini desktop computers connected to 17 inch CRT monitors, refreshed at a rate of 85 Hz. Participants made their responses using a standard Apple keyboard and mouse. Testing was conducted in individual cubicles in groups of up to five, with sound feedback delivered via personal headphones.

#### Procedure

In the training phase, participants were asked to assume the role of a pharmaceutical researcher whose job was to determine the effects of different drugs using trial and error. On each trial a drug or combination of drugs was presented and participants were asked to predict which of three possible outcomes they thought might occur (migraine, nausea or no outcome) by clicking on one of the buttons below the drug names. When an answer was selected, the box surrounding the outcome turned yellow, the three buttons disappeared and were replaced by the correct answer while the drug names remained on the screen. The drug names appeared in one of 3 colours (blue, green or red) and either a picture of a sad face or medicine was displayed on the feedback screen if the correct outcome was one of the illnesses. The choice of cue colour and picture was not systematically related to particular cues or outcomes.

Participants in the accuracy group were told to do the task as accurately as they could and to take their time, receiving a buzzer tone and the word ‘INCORRECT’ on the top of the screen if they made an error, as well as the word “correct” in smaller font if they chose correctly. Participants in the speed group were told to complete the task as fast as they could and were given only 1.5 seconds to respond, after which a buzzer tone was heard and the word ‘FASTER’ appeared at the top of the screen and no response recorded. The speed group were not given any feedback as to whether they were correct or incorrect and were only shown the correct answer. All contingencies were consistent throughout and therefore each stimulus presentation fully predicted a particular outcome. There were 8 blocks of 24 trials presented continuously without break for the entire training phase (192 trials in total). Within each block there were 2 repetitions of the 12 trial types (see Table 1), with their order of appearance randomised within each block. The spatial presentation of stimuli within each compound was counterbalanced so equal numbers of each were seen (e.g. AX and XA).

In the ratings test, participants were asked to rate the likelihood of each of the two outcomes occurring given the presence of one or two of the drug cues. On each trial, the drug name(s) appeared at the top of the screen, followed by two linear analogue scales appearing next to each of the outcome names (i.e. one scale for migraine, one for nausea). The end points of each scale were labelled “definitely will not occur” to “definitely will occur”. Participants could click anywhere on the scale, yielding ratings ranging from 0–100. The order of presentation was randomised, with each single-cue stimulus presented once, and each compound twice, again with the order of presentation within each compound counterbalanced. The ratings test was self-paced.

The last phase of the experiment (the inference test) aimed to extract a rational predictive judgement about the test stimuli by presenting all the relevant contingencies in the summation test at once on the screen. Participants were told that they would be viewing the results of the drugs again and could make another reasoned judgement which could be the same or different as before. Participants were shown that A_1_ led to outcome 1, A_1_X_1_ led to no outcome, B_1_ led to outcome 1 and C_1_ led to no outcome (A_1_+/A_1_X_1_−/B_1_+/C_1_−). They were then asked to rate how likely both outcomes 1 and 2 were to occur for the compounds B_1_X_1_ and B_1_C_1_ (the same compounds used in the summation test). These ratings were made in the same fashion as the predictive ratings, with all scores transformed to a scale of 0–100. This was then repeated for the corresponding stimuli with outcome 2 (A_2_+/A_2_X_2_−/B_2_+/C_2_−, test C_2_X_2_ and C_2_E_2_). All drug name allocations and drug-illness contingencies were the same as in training, with all writing presented in white on a black background.

### Results and Discussion

All analyses were performed with an alpha level of.05 and Greenhouse-Geisser adjusted p-values are reported where relevant.

#### Training

**Figure 1:**
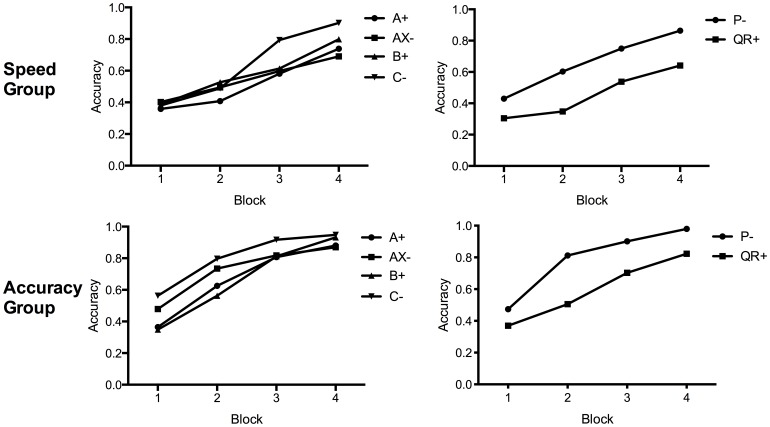
Training accuracy in Experiment 1 over blocks. Averaged over trial type (i.e. A_1_ and A_2_ averaged into A). Upper panels: speed group, lower panels: accuracy group. Left panels: feature negative stimuli and controls, right panels: filler stimuli.

Figure 1 shows accuracy for each stimulus type across training, averaged in four equal blocks. Over all stimuli, the accuracy group were more accurate throughout all training blocks, lowest *F*(1, 46) = 5.09, *p* = .029, and overall, *F*(1, 46) = 18.32, *p*<.001. As expected, the speed group responded faster overall, *F*(1, 46) = 61.78, *p*<.001, by a mean of 1.00 seconds per trial. Even in the last 24 trials when the accuracy group were at their best performance (about 90.5% correct), they were still slower than the speed group by 0.50 seconds, *F*(1, 46) = 28.51, *p*<.001. Thus the pacing and instructions succeeded in manipulating both accuracy and the time spent on each trial.

#### Ratings test

Table 2 shows the ratings given for all stimuli tested for both groups. For the summation test, variables were computed to represent the mean of the test stimuli (B_1_X_1_ and B_2_X_2,_ henceforth BX) and the mean of the control stimuli (B_1_C_1_ and B_2_C_2_, henceforth BC) as difference scores (O1–O2 for B_1_X_1_ and B_1_C_1_, O2–O1 for B_2_X_2_ and B_2_C_2_), and as outcome-specific scores (O1 for B_1_X_1_ and B_1_C_1_, O2 for B_2_X_2_ and B_2_C_2_).

**Table 2:** Ratings for both outcomes in Experiment 1 (distractors omitted).

	Speed Group		Accuracy Group	
Test Cues	O1 Rating	O2 Rating	O1 Rating	O2 Rating
B_1_X_1_	47.4	25.4	45.3	20.8
B_2_X_2_	23.4	45	27.4	50.3
B_1_C_1_	38.2	18	51.6	16.5
B_2_C_2_	17.1	36.2	17.9	49.9
A_1_	67.3	11.4	71.7	12.8
A_2_	27.3	60.6	10.8	86.2
A_1_X_1_	35.5	18.6	24	11.3
A_2_X_2_	20.6	31.9	15.3	19
B_1_	72.5	12.6	84.4	16
B_2_	17.3	68.7	9.2	91.1
C_1_	16.3	15.3	5.1	10.6
C_2_	18.9	16	8.1	7.3
X_1_	36.6	26.9	12.5	11.5
X_2_	29.9	28.1	22.8	29.2

All compound cues presented twice (e.g. B_1_X_1_ and X_1_B_1_) and all single cues presented once (e.g. X_1_).

**Figure 2:**
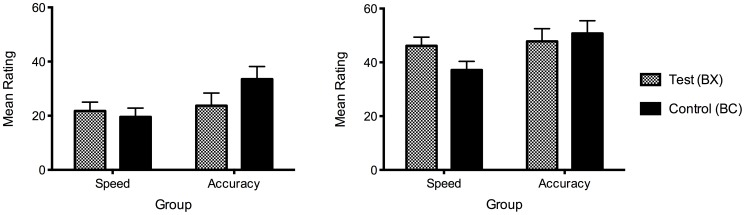
Summation tests comparing control stimuli (BC) against test stimuli (BX) for both outcomes. Left panel: difference scores, calculated as the difference between O1 and O2 ratings for B_1_X_1_ and B_1_C_1_, and the difference between O2 and O1 ratings for B_2_X_2_ and B_2_C_2_. Right panel: outcome-specific scores, using the rating for O1 only for B_1_X_1_ and B_1_C_1_, and the rating for O2 only for B_2_X_2_ and B_2_C_2_. Error bars represent the standard error of the mean difference between test and control.

For both these scores, in the accuracy group, the test stimuli were rated lower than the controls, whereas the reverse was found in the speed group (Figure 2). Two separate 2×(2) repeated measures ANOVAs with group as the between-subjects factor comparing the differences between test and control found a significant interaction with group using both the difference scores, *F*(1, 45) = 4.40, *p = *.042, and the outcome-specific scores, *F*(1, 45) = 4.32, *p = *.043 (Figure 2).

To detect the presence of second-order conditioning in the speed group and conditioned inhibition in the accuracy group, the difference between the test (BX) and control (BC) stimuli was analysed for each group separately. Using the difference scores, there was no significant difference between the test and control stimuli for the speed group, *F*<1, but within the accuracy group, the test stimuli were rated significantly lower than the controls, *F*(1, 22) = 4.43, *p = *.046, consistent with conditioned inhibition (Figure 2, left panel). This meant that participants given instructions emphasising accuracy showed a reduced preference for predicting the outcome associated with the excitor relative to the unrelated outcome. Using the same analysis on the outcome-specific scores, evidence of second-order conditioning was found in the speed group with the test stimuli rated significantly higher than the controls, *F*(1, 22) = 7.83, *p = *.010 (Figure 2, right panel), but no evidence of conditioned inhibition in the accuracy group, *F*<1. This indicates that for the speed group, there was a general inflation of prediction ratings for both outcomes in the presence of the test stimulus rather than an increased preference towards predicting its related outcome.

Thus the main hypothesis that different instructions and trial timeouts emphasising speed or accuracy could produce opposing patterns of learning was supported, with the speed group displaying second-order conditioning and the accuracy group showing conditioned inhibition. Notably, group interactions were obtained on both summation test measures, extending the findings of Karazinov and Boakes [17], who detected second-order conditioning and conditioned inhibition across experiments but failed to find a significant interaction of the summation test with group. It is interesting that conditioned inhibition was found with the difference scores, a measure more sensitive to outcome specificity, and second-order conditioning was found on the outcome-specific scores where the unrelated outcome was not considered. This finding is perhaps a reflection of the nature of these learning effects. The learned properties of a conditioned inhibitor are thought to be bound to the outcome associated with the paired excitor [3], which may explain why conditioned inhibition manifested on the difference scores. Meanwhile second-order conditioning may have emerged in the speed group because participants regarded×as contributing to the likelihood of a side-effect occurring but could not remember which outcome this test stimulus had been paired with (in fact, it was never paired directly with either migraine or nausea). Hence, the observed effect was to inflate ratings of BX relative to BC on both outcome scales.

It is also worth noting that while all participants rated an unambiguously causal cue (B) quite high, and an unambiguously non-causal cue (C) quite low (see Table 2), ratings to the control compound BC were substantially lower than to B, indicating a strong generalization decrement resulting from the addition of a non-causal cue. Additional ANOVAs were used to compare the trained cues (B_1_/B_2_) against the summation controls (B_1_C_1_/B_2_C_2_), using the outcome-specific scores (O1 for B_1_ and B_1_C_1_, O2 for B_2_ and B_2_C_2_) in one analysis and the difference scores (O1–O2 for B_1_ and B_1_C_1_, O2–O1 for B_2_ and B_2_C_2_) in the other. Both analyses yielded significantly higher scores for B than for BC (smaller *F*(1,44) = 51.08, p<.001), but neither revealed an interaction with group (*F*s<1), suggesting that the amount of generalization decrement resulting from the addition of the non-causal cue C was very similar in the two groups. While the ratings for BC seem to differ between groups, this is probably due to the fact that B itself received different ratings from the two groups.

#### Inference test

**Figure 3:**
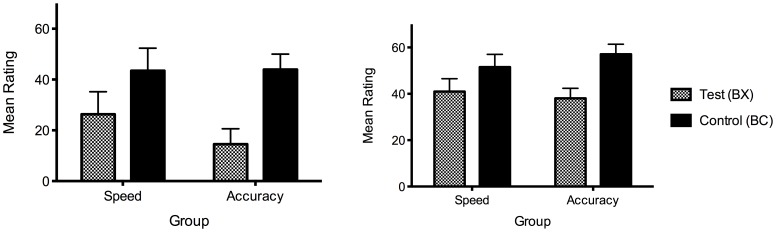
Results from summation test stimuli presented again during inference test. Presented A+/AX−/C+/DE-, tested CX, CE, for both outcome 1 and 2. Left panel: difference scores, calculated as the difference between O1 and O2 ratings for B_1_X_1_ and B_1_C_1_, and the difference between O2 and O1 ratings for B_2_X_2_ and B_2_C_2_. Right panel: outcome-specific scores, using the rating for O1 only for B_1_X_1_ and B_1_C_1_, and the rating for O2 only for B_2_X_2_ and B_2_C_2_. Error bars represent the standard error of the mean difference between test and control.

Due to its similarity to the ratings test, the data from the inference test were analysed in the same way, with variables computed to represent the average of the test stimuli and the controls, using both difference scores and outcome-specific ratings. A 2×(2) repeated measures ANOVA on the difference scores (Figure 3, left panel) revealed a significant difference between the test stimuli and the controls, *F*(1,45) = 19.19, *p*<.001, and this did not interact with group, *F*<1. A 2×(2) repeated measures ANOVA was also conducted on the outcome-specific scores, yielding a significant difference between the test and control stimuli, *F*(1,45) = 18.21, *p*<.001, but again no interaction with group, *F*(1,45) = 1.49, *p = *.229 (Figure 3, right panel). Thus neither analyses revealed any group differences on the inference test, with both groups rating the test compounds lower than the controls, consistent with conditioned inhibition.

The above results indicate that both groups judged the test stimuli as inhibitory given the chance to reflect on, and make a rational inference about, the contingencies. This suggests that speeded learning conditions can result in learning that is non-rational. While this does not necessarily entail that learning was driven purely by associative mechanisms, it does suggest that a type of learning that is better explained by associative theory emerges when people learn under strict time constraints, processing information quickly and without careful thought. This experiment also shows that the speed group switched to judging the test stimuli as inhibitory once given the chance to reflect carefully on the contingencies, suggesting that second-order conditioning is not readily explained by a reasoned inference. Since the current manipulation was successful in dissociating learning in the FN paradigm, Experiment 2 sought to replicate this dissociation and examine its relationship with other cue competition effects that are thought to be mediated by cognitive resources.

## Experiment 2

The results of Experiment 1 support Karazinov and Boakes’ [17] conclusion that limiting time to think during learning a FN discrimination may yield the conditions necessary to observe second-order conditioning rather than conditioned inhibition. Experiment 2 sought to replicate the dissociation found in Experiment 1, provide stronger evidence of second-order conditioning and conditioned inhibition using the same test measures, and examine whether other seemingly irrational learning effects could also be obtained using the speed manipulation. Recently, Vadillo and Matute [19] described such an effect using a blocking design. Blocking occurs when a target cue is paired with an outcome, but is always presented in compound with another cue that has previously been established as a strong predictor of that outcome. Typically, the target cue is rated as being less likely to cause the outcome than control cues that are only trained in compound. Thus, learning about the target cue is blocked by the presence of a strong predictor. This blocking effect is routinely observed in animal conditioning and, under many conditions, is also reliably found in causal learning experiments (e.g. [24], [25]). However, under time pressure, Vadillo and Matute observed augmentation rather than the typical blocking effect, where the target cue was given a higher rating than the control cues. This is a striking finding as it is not easy to explain in terms of either rational inference or associative learning principles, which normally predict blocking. Although, in theory, the target cue could acquire some excitatory strength via the within-compound association, demonstrations of augmentation are rare in the animal and human learning literature.

The time-to-think hypothesis proposed by Karazinov and Boakes [17] is also clearly relevant to another set of cue competition effects – retrospective revaluation effects – that are assumed to require the retrieval of learned cues in order for revaluation of learning to occur. An example relevant to the current design is release from overshadowing; after a compound of two cues are paired with the outcome, one cue is then presented individually and is shown not to cause the outcome. The individual presentations of this cue affect the ratings of the target cue that is not presented, relative to controls that are simply trained in compound. Typically, participants rate the target cue as being more likely to cause the outcome than the controls [26]. However, presentation of the non-causal cue in isolation can sometimes have the opposite effect on ratings of the target cue, an effect called mediated extinction. Using an AB+/A− design, Liljeholm & Balleine ([18], Experiment 1) found that encouraging the amount of generalization that occurred between A and B resulted in judgments consistent with mediated extinction. This was achieved by using visual cues that were joined spatially so as to encourage configural processing. Their study sheds some light on why mediated extinction is usually found in animal studies where the cues consist of flavours and odors (e.g. [27]), and release from overshadowing found in causal judgement tasks, where participants are more likely to treat the cues as independent causes of the outcome. As with second-order conditioning and augmentation, it seems difficult to account for mediated extinction in terms of a logical inference. But in this case, the associative explanation of this seemingly irrational effect relies upon inhibitory learning (or the weakening of associations) during the extinction of A- being transferred to B through within-compound associations. Both release from overshadowing and mediated extinction require retrospective revaluation of a cue that is not presented and therefore rely to some extent on retrieval of cues on the basis of within-compound associations.

In Experiment 2, the FN contingencies were retained and new contingencies were added to assess retrospective revaluation and blocking. Retrospective revaluation was examined using a blocked feature positive (FP) discrimination (FY+ followed by F−) where the critical revaluation effects were revealed by comparing ratings of the test cue Y with ratings of control cues (G and H) that were previously trained in compound (GH+). Learning about a redundant cue was examined using the conventional forward blocking design in which one of two cues is pretrained (I+) and followed by compound training with the target cue (IZ+).

Training in Experiment 2 was divided into two phases with the FP and blocking stimuli presented in separate phases, and the FN stimuli presented consistently throughout (see Table 3 for design). All other training parameters and instructions were the same as in Experiment 1. Due to the increased complexity of the design, the second outcome was omitted to reduce the total number of training stimuli so that the only outcomes were migraine (+) and no outcome (–). Karazinov and Boakes [17] used a control compound consisting of a trained excitor and a control cue (TM) where M was trained in compound (LM−), which differs slightly from the control used in Experiment 1, where we combined a trained excitor with a control cue (C−) which unambiguously predicted no outcome. While they were very similar, the subtle differences between these controls may prove to be important and as such, two control compounds were included in Experiment 2: BC, as used in Experiment 1, and BE where E was previously non-causal but trained in compound (DE−).

**Table 3:** Cues used in the training phase and ratings test of Experiment 2.

	Phase 1	Phase 2	Ratings Test
FN stimuli	A+	A+	BX vs. BC
	AX−	AX−	BX vs. BE
Controls	B+	B+	
	C−	C−	
	DE−	DE−	
FP stimuli	FY+	F−	Y vs. G/H
Controls	GH+		
Blocking Stimuli	I+	IZ+	Z vs. J/K
Controls		JK+	

Phase 1 contained 5 blocks, phase 2 contained 3 blocks. Outcomes were either migraine (+) or no outcome (−).

### Method

#### Participants

Eighty-four first-year psychology students from the University of Sydney participated in exchange for partial course credit. Participants who scored below 55% accuracy (again, slightly above chance) for either or both of the last quarters in each training phase were excluded, resulting in four exclusions, all from the speed group. Two further participants (one in each group) were excluded from analysis because they scored more than 3 standard deviations from the mean on the critical test scores, leaving 38 participants in the speed condition, and 40 in the accuracy condition (49 female, mean age = 19.78 years).

#### Apparatus

The apparatus used was identical to that in Experiment 1.

#### Procedure

The procedure was identical to that in Experiment 1 except for the following changes.

Unlike Experiment 1, training now consisted of 2 separate phases containing different stimuli (see Table 3). The first phase had 5 blocks of 32 trials and the second phase had 3 blocks (256 trials in total). Within each block, each of the 8 trial types was repeated 4 times, and there was a short rest period after 4 blocks. Since outcome 2 was removed in Experiment 2, participants made ratings on just the one scale (migraine) in the ratings test.

### Results and Discussion

#### Training

**Figure 4:**
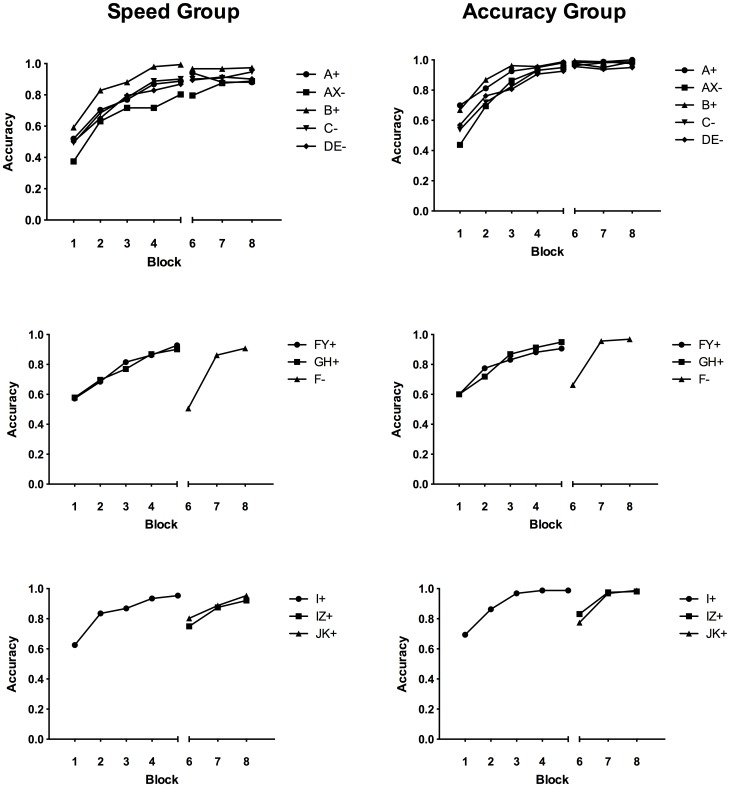
Training accuracy over blocks divided into FN (top), FP (middle) and blocking stimuli (bottom). Left panel: speed group, right panel: accuracy group. Phase 2 began at block 6.

The accuracy group were significantly slower and more accurate in both training phases, and overall, lowest *F*(1, 76) = 9.59, *p* = .003. On average, the accuracy group took 0.56 seconds longer than the speed group in the first phase, and 0.97 seconds longer in the second phase. Figure 4 shows training accuracy over both phases for both groups.

#### Ratings test

Table 4 shows the ratings for all test cues across both groups. Statistical analyses were performed on each of the three cue learning effects individually.

**Table 4:** Ratings for all test cues in Experiment 2.

	Speed Group	Accuracy Group
A	92.4	89.8
AX	9.6	15.3
B	90.2	89.9
BX	58.2	44.6
BC	45	46.3
BE	46	52.6
F	21.1	9.4
Y	62.8	64.6
G	65.6	59.6
H	68.6	62.4
I	88	90
Z	67.8	70.5
J	75.7	72.8
K	67.9	64.6

All compound cues presented twice (e.g. BX and XB) and all single cues presented once (e.g. X).

**Figure 5:**
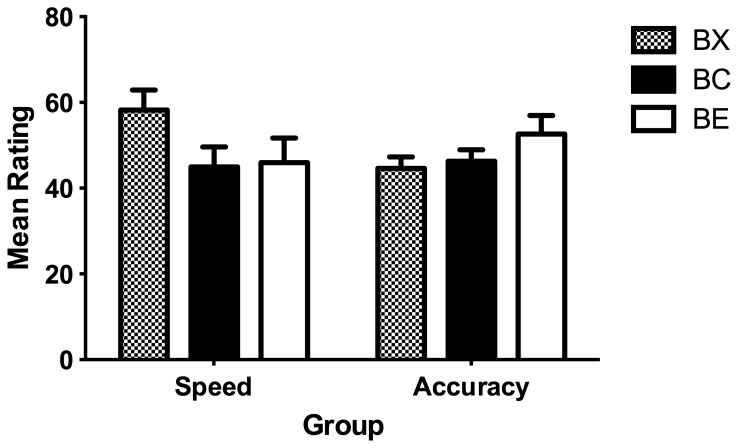
Summation tests for Experiment 2 comparing the test stimulus (BX) against controls (BC, middle, and BE, right). Scores indicate ratings made for the single outcome (migraine) in Experiment 2. Error bars represent the standard error of the mean difference between BX and each of the controls.

Using the summation test used by Karazinov and Boakes [17] comparing BX to BE in the feature negative discrimination, there was no difference between test and control stimuli, *F*(1, 76) = .27, *p = *.60, but this interacted with group, *F*(1, 76) = 8.11, *p* = .006. In the speed group, there was evidence of second-order conditioning, *F*(1, 37) = 4.65, *p = *.038, with higher ratings to BX than to BE, while conditioned inhibition in the accuracy group was marginally non-significant, *F*(1, 39) = 3.38, *p* = .074, with lower ratings to BX than to BE (see Figure 5). Thus the group interaction and strong second-order conditioning effect in the speed group were again evident, with results also suggestive of a conditioned inhibition effect in the accuracy group.

Similar results were also found with the summation test comparing BX to BC (Figure 5), with a significant group interaction, *F*(1, 76) = 7.93, *p* = .006, evidence of second-order conditioning in the speed group with higher ratings for BX than BC, *F*(1, 37) = 8.12, *p* = .007. However, in the accuracy group, there was no evidence of conditioned inhibition when comparing BX to BC, *F*<1. This replicates the results from the outcome-specific scores in Experiment 1, where a significant group interaction and second-order conditioning was found, but no conditioned inhibition. Using either the BE or BC control, there is a clear group interaction, suggesting that forcing participants to adopt a speed strategy during training affects what is learned in the FN discrimination. However, the results highlight the subtle differences between using non-causal control cues trained in compound or individually. It may have been that because C was unambiguously non-causal, ratings to BC were lower than ratings to BE, thus making it harder to detect conditioned inhibition when comparing BX to BC.

For the stimuli used in the blocked feature positive discrimination, there were no overall differences between test (Y) and control stimuli (G/H), *F*<1, and no interaction with group, *F*(1, 76) = 1.43, *p* = .235, although the effects were in the hypothesized direction, with the speed group rating the test stimuli lower than the controls (62.8 vs. 67.1), and the accuracy group showing the reverse pattern (64.6 vs. 61.0).

Similarly, for the stimuli used in the blocking contingencies, there were no overall differences between test (Z) and control stimuli (J/K), and no interaction with group, larger *F*(1, 76) = 1.32, *p* = .255. Although far from being statistically significant (*F*s<1), ratings for the blocked cue were slightly lower than the overshadowing control cues in the speed group (67.8 vs. 71.8) but slightly higher than the overshadowing control cues in the accuracy group (70.5 vs. 68.7). Thus, there is certainly no evidence of a greater propensity towards augmentation in the speed group.

**Figure 6:**
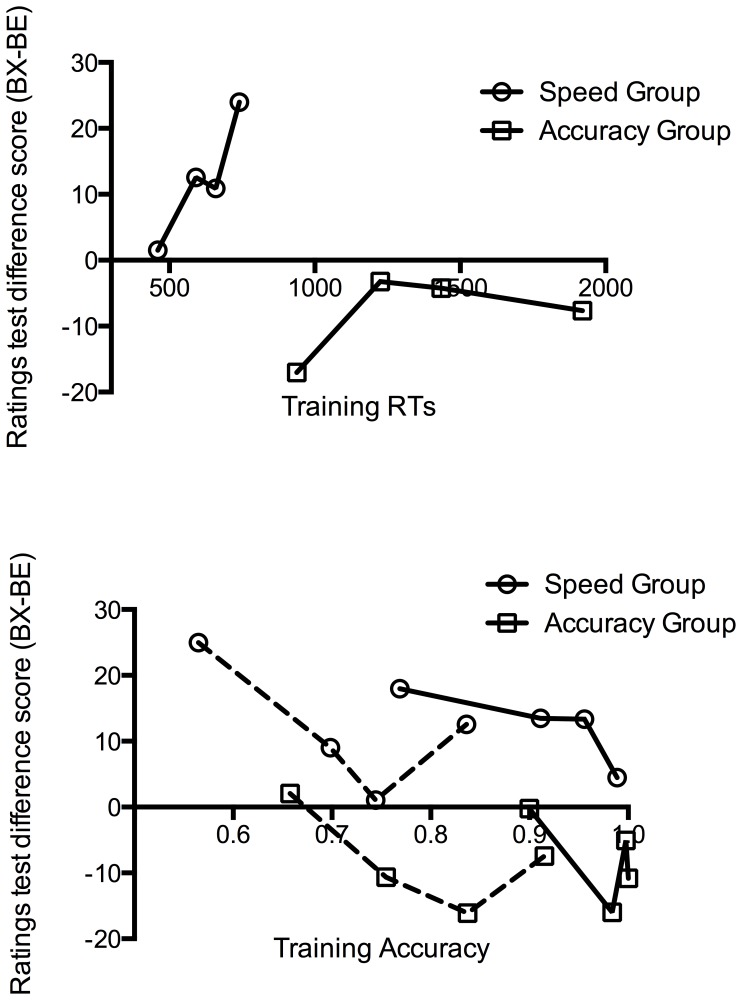
Summation test difference scores (BX-BE) in Experiment 2 separated into ranked quartiles according to training performance. Upper panel: Ratings ranked according to mean reaction time (in ms) during training. Lower panel: Ratings ranked according to mean training accuracy in the first half (dotted lines) and second half (solid lines) of training.

As noted, participants in the speed group completed training faster but also less accurately than participants in the accuracy group. One explanation for the group interactions is that the degree of conditioned inhibition or second-order conditioning has a simple and direct relationship to how well the FN discrimination and related control trials were learned. That is, the speed group merely showed second-order conditioning because they did not learn these trials well. Alternatively, speeding the trials may have changed the way that participants were engaging in the learning task. To address this, we examined performance on the BX vs. BE ratings difference when participants in each group were split into quartiles according to their training performance for all FN paradigm trials (A+, AX−, B+, C−, DE−). This quartile analysis is shown in Figure 6, conducted separately for each group and for each of a) mean reaction time across all training trials, b) early training accuracy (across the first half of the training phase), and c) late training accuracy (across the second half of the training phase). Both early and late training analyses are included simply because although accuracy near the end of training might well be expected to closely predict test performance, most of the accuracy group were near ceiling over the second half of training. A clear group division is apparent in the speed with which the task was performed, with the slowest quarter of the speed group still substantially faster than the fastest quarter of the accuracy group (Figure 6, upper panel). In terms of the BX-BE difference score, there is also a clear division, with all quarters of the accuracy group showing a negative score and all quarters of the speed group showing a positive score. Within the speed group, there is also some indication that faster participants showed less of a difference between BX and BE, possibly suggesting that those participants who were better able to perform under the task demands were less likely to show second-order conditioning (the fastest participants were also more accurate than the rest of the sample, 0.86 vs. 0.79).

Examining the division in terms of accuracy, the upper three quarters of participants in the speed group overlapped with the lower three quarters of participants in the accuracy group, both early and late in training (Figure 6, lower panel). It appears that in both groups and for both early and late training rankings, the participants who performed the worst (i.e. the lowest quarter) displayed higher ratings for BX relative to BE than the other participants within their respective group. This and the general pattern across the quartiles suggests that participants who struggled most with learning the relevant contingencies also displayed more second-order conditioning. However there is still a clear division in BX-BE difference scores between the groups at each quartile. The top three quarters of the speed group have equivalent training accuracy to the bottom three quarters of the accuracy group when ranked according to early training accuracy (76.3% vs. 75.0%, *t*(56) = 0.63, *p* = 0.53) or late training accuracy (95.3% vs. 96.0%, *t*(56) = 0.52, *p* = 0.60). Nevertheless, these curtailed samples differ significantly in the BX – BE ratings difference for both the early training ranks (7.72 vs. −8.23, *t*(56) = 2.09, *p* = 0.04) and late training ranks (10.2 vs. −7.1, *t*(56) = 2.29, *p* = 0.03). Thus even participants in the speed group who performed with equivalent accuracy to those in the accuracy group still rated the FN stimuli very differently, showing more evidence of second-order conditioning. Equivalent accuracy during training does not imply equivalent levels of learning because the training predictions of the speed group were made under time constraints, meaning that the lower accuracy in this group may partly reflect a trade-off with decision speed. Therefore, if anything, training accuracy in the speed group probably underestimates true learning relative to the accuracy group. If the BX-BE difference were a simple monotonic function of how well the contingencies were learned, then one would have to assume that the speed group accuracy substantially *overestimated* the extent of learning relative to the accuracy group. Therefore, it seems that participants in the speed group learned about the FN contingencies differently to the accuracy group, and that the difference is not merely a consequence of poorer learning per se.

In summary, Experiment 2 replicated the dissociation found in Experiment 1 using two different summation tests but did not find dissociations in either retrospective revaluation or learning about a redundant cue. When each group was separated according to training performance, the group differences in conditioned inhibition and second-order conditioning were evident across overlapping levels of training accuracy, suggesting that the effect of speeding the training trials was not merely driven by poor learning in the speed group. Rather, it suggests that this group learned something qualitatively different about the FN discrimination.

## General Discussion

Over both experiments, paced training, with instructions and feedback emphasising speed, resulted in second-order conditioning rather than the usual result of conditioned inhibition in the FN discrimination. Unpaced training, with instructions and feedback emphasising accuracy, resulted in learning that was consistent with conditioned inhibition, although several of the simple effects in both experiments failed to reach significance. Importantly, group interactions were found in Experiment 1 with two measures of learning – outcome-specific ratings and difference scores – and with two subtly different summation test controls in Experiment 2. The inference test in Experiment 1, in which both groups rated the test cues (X_1_ and X_2_) as if they were preventative, suggests that the second-order conditioning observed in the speed group cannot readily be explained as a reasoned or rational inference, as they showed an inhibitory judgement in the inference test but a causal one in the ratings test. In Experiment 2, the same manipulation that produced the dissociation in the FN paradigm was not successful in dissociating retrospective revaluation effects (release from overshadowing versus mediated extinction) or learning about a redundant cue (blocking versus augmentation). Experiment 2 did not replicate Vadillo and Matute’s [19] finding of time pressure producing augmentation. This might be due to differences in test procedures; causal ratings were used in Experiment 2 whereas an online response measure within a restricted time limit was used in Vadillo and Matute’s study.

While this study shows that the dissociation found by Karazinov and Boakes [17] is replicable, the task of finding the appropriate experimental parameters to reveal both second-order conditioning and conditioned inhibition is clearly a challenging one. In the present study, group interactions and second-order conditioning were present in both experiments, with the conditioned inhibition effect in Experiment 2 approaching significance. At the very least, this suggests that second-order conditioning emerges when learning conditions are speeded, and not when participants are advised to think carefully about their responses and reason through the contingencies. One reason for the weak evidence for conditioned inhibition may be due to the large amount of individual variability when training is unpaced. In Experiment 1, conditioned inhibition was observed only on a ratings difference score that gauges learning about the specific outcome associated with the excitatory test cue, and in Experiment 2, the conditioned inhibition effect only approached statistical significance. Karazinov and Boakes highlight that there are substantial individual differences in learning the FN discrimination, arguing that some participants are more disposed than others to deliberating about the logical relationships involved. These participants might be expected to learn the inhibitory relationship faster than others, regardless of the group to which they were allocated. Likewise, while the feedback and timeout were successful in forcing the speed group to respond more quickly, there might well be considerable variation in the degree to which participants adhere to instructions emphasising the importance of accuracy, and the effect these instructions have on the manner in which they go about the task. Nevertheless, across the two experiments, we replicated the summation test group interactions on all four measures of conditioned inhibition and, just as importantly, found clear evidence of second-order conditioning under paced conditions. The quartile analysis of the Experiment 2 results also suggests that the group difference in producing second-order conditioning versus conditioned inhibition is fairly consistent across different levels of individual performance during training.

Associative learning theory can account for the second-order conditioning observed in these experiments in much the same way as for the equivalent results in the animal learning literature. To this end, one must assume that the within-compound association between A and×mediates an initial excitatory relationship between×and the outcome, until participants gradually learn the inhibitory association between×and the outcome. This suggests that learning about×passes through an initial excitatory stage before reaching inhibition, although does not reveal whether this mediated excitation is eliminated or simply masked by inhibitory learning. In any case, it implies that excitatory and inhibitory learning are closely linked, with excitation a prior and perhaps necessary stage of learning that must occur before conditioned inhibition develops [17]. From this perspective, the question then remains: in what fashion does trial pacing prevent or delay the formation of an inhibitory relationship between×and the outcome?

Forcing participants to learn in a speeded way appears to have the same effect as reducing the length of training in rats [15]. Limiting the time to make decisions on each trial might simply lower the learning rate in a general sense, thus providing a greater opportunity to observe the early stages of cue learning in which second-order conditioning is more apparent. A general slowing of the learning rate seems more likely than, for instance, a selective impairment of inhibitory learning given the results of Experiment 2. If anything, the speed group showed slightly more tendency towards mediated extinction. However, it is clear that a substantial proportion of the speed group learned the FN discrimination fairly well and yet still showed greater evidence for second-order conditioning than the accuracy group. It should also be noted that time constraints were only implemented over the initial part of each trial leading up to the participant’s prediction and thus both the speed and accuracy groups observed the cues paired on screen with the appropriate outcome for exactly the same length of time. This implies that the time during which participants make a prediction was critical in determining whether conditioned inhibition or second-order conditioning was observed.

This suggests that perhaps simply encoding the cue-outcome relationships may not be sufficient for conditioned inhibition to occur. Pacing each trial may impede the formation of a prediction about the occurrence of the outcome, or prevent generalization of a learned prediction to similar trial types. For instance, participants may learn the association between cue A and the outcome. However, on AX- trials, prediction error might be reduced because participants are unable to form a strong prediction on the basis of the A-outcome association due to the trial time limit. Unlike the accuracy group, the feedback presented to the speed group on each trial did not emphasise when an error was made, which may have also served to reduce the impact of prediction error on learning. The feedback, instructions or pacing may have been critical in producing the dissociation in our results. The combination of these parameters in the speed group clearly does not provide the circumstances necessary for×to develop strong inhibition. On test, when the participants’ decisions are no longer paced, the excitatory association mediated through the within-compound relationship between A and×may have a greater influence on their ratings, leading to second-order conditioning.

On the other hand, the time restrictions may have removed the opportunity or motive for the participant to think about the structure of the task in more cognizant or deliberative fashion, thereby preventing the inference that cue×was preventative. This propositional account implies that many observations of conditioned inhibition in human causal learning – observed under similar unpaced conditions and across procedures of similar duration – are probably not indicative of the gradual development of an inhibitory association. However, the inference test from Experiment 1 suggests that judgements of second-order conditioning are not readily obtained and not easily explained by propositional reasoning. Therefore, a possible explanation is that excitatory learning mediated by the causal cue A can be masked or overridden by a reasoned inference about X. This argument is similar to that proposed by Mitchell, Livesey and Lovibond [12], who used a feature negative discrimination with two outcomes (A+1/BX−/B+2/BY−) in an unpaced learning task. They found that contingency judgements revealed inhibitory learning between×and outcome 1, and Y and outcome 2, but×was more easily categorized with outcome 1 and Y with outcome 2 on a speeded categorization task, indicating the existence of an excitatory association. Mitchell et al. thus concluded that it is possible to learn an excitatory relationship yet express an inhibitory one. They further suggested that learning of the preventative relationship between inhibitor and outcome required an extra inferential step in addition to knowing that× “went with” outcome 1.

Mitchell et al.’s [12] conclusion that learned associations do not always translate directly into contingency judgements is reconcilable with the present study if we assume that while the accuracy group did learn and express the inhibitory relationship in their causal ratings, they may have also learned and retained the second-order relationship. Meanwhile, the speed group may have been impeded in their acquisition of the inhibitory relationship and thus expressed a causal relationship×and the outcome, mediated by the causal cue A. The present study however, cannot attest to whether the inhibition displayed by the accuracy group in the ratings test was acquired during training through an inference or the gradual formation of associative links. Thus our results are consistent with the broader assertion that inhibitory learning in causal judgement tasks might be based on reasoning rather than the formation of an inhibitory link, a possibility raised by both Mitchell et al. and Karazinov and Boakes [17]. However, the task of dissociating this from an associative explanation is not trivial, particularly since associative theories do not mandate that excitatory and inhibitory associations are mutually exclusive. Regardless of the mechanism responsible for inhibitory learning, the fact remains that second-order conditioning occurs reliably when participants’ time to think is restricted during learning, and does not occur when deliberation is encouraged.

In conclusion, the current results add further evidence of a second-order conditioning effect in human causal learning when participants are given only a limited time to make predictions during training. Learning about a preventative cue was consistently influenced by trial timing and instructions that emphasized either speed or accuracy. This effect may well prove to be diagnostic of the mechanisms that contribute to human causal learning. Given its potential for distinguishing theoretical accounts of causal learning, it certainly warrants further investigation.
